# Supplementary material for: Combinatorial Gene Regulatory Functions Underlie Ultraconserved Elements in Drosophila
Source: Mol Biol Evol. 2016 May 31;33(9):2294–306. doi: 10.1093/molbev/msw101 (PMC4989106; doi:10.1093/molbev/msw101)
Supplement: Supplementary Data [file supp_33_9_2294__index.html]

Combinatorial gene regulatory functions underlie ultraconserved elements (UCEs) in Drosophila — Combinatorial Gene Regulatory Functions Underlie Ultraconserved Elements in Drosophila — Combinatorial Gene Regulatory Functions Underlie Ultraconserved Elements in Drosophila — Supplementary Data 

# Combinatorial Gene Regulatory Functions Underlie Ultraconserved Elements in Drosophila

## Supplementary Data

files

- Supplementary Data - zip file
