# Supplementary material for: Combinatorial Gene Regulatory Functions Underlie Ultraconserved Elements in Drosophila
Source: Mol Biol Evol. 2016 May 31;33(9):2294–306. doi: 10.1093/molbev/msw101 (PMC4989106; doi:10.1093/molbev/msw101)
Supplement: Supplementary Data [file supp_msw101_suppl_data.zip › Warnefors et al_TEXT_S04_Clean.docx]

*Article*

**Combinatorial gene regulatory functions underlie ultraconserved elements (UCEs) in Drosophila**

Maria Warnefors^1,2,3,4,*,†^, Britta Hartmann^5,*^, Stefan Thomsen^1^ and Claudio R. Alonso^1,†^

*(1) Sussex Neuroscience, School of Life Sciences, University of Sussex, Brighton BN1 9QG, United Kingdom*

*(2) Center for Integrative Genomics, University of Lausanne, 1015 Lausanne, Switzerland*

*(3) Swiss Institute of Bioinformatics, 1015 Lausanne, Switzerland*

*(4) Present address: Zentrum für Molekulare Biologie der Universität Heidelberg (ZMBH), Im Neuenheimer Feld 282, 69120 Heidelberg, Germany*

*(5) University Medical Center Freiburg, Institute of Human Genetics, 79102 Freiburg, Germany and BIOSS* *Centre for Biological Signaling Studies, Schänzlestrasse 18, 79104 Freiburg, Germany*

*(*) Equal contribution*

*(†) Corresponding authors:*

**Maria Warnefors**

Tel +41 21 692 3962

Email [maria.warnefors@gmail.com](mailto:maria.warnefors@gmail.com)

**Claudio R. Alonso**

Tel +44 1273 876621

Email [c.alonso@sussex.ac.uk](mailto:c.alonso@sussex.ac.uk)

**ABSTRACT**

Ultraconserved elements (UCEs) are discrete genomic elements conserved across large evolutionary distances. Although UCEs have been linked to multiple facets of mammalian gene regulation their extreme evolutionary conservation remains largely unexplained. Here we apply a computational approach to investigate this question in Drosophila, exploring the molecular functions of more than 1,500 UCEs shared across the genomes of twelve Drosophila species. Our data indicate that Drosophila UCEs are hubs for gene regulatory functions and suggest that UCE sequence invariance originates from their combinatorial roles in gene control. We also note that the gene regulatory roles of intronic and intergenic UCEs (iUCEs) are distinct from those found in exonic UCEs (eUCEs). In iUCEs, transcription factor (TF) and epigenetic factor binding data strongly support iUCE roles in transcriptional and epigenetic regulation. In contrast, analyses of eUCEs indicate that they are two orders of magnitude more likely than the expected to simultaneously include protein-coding sequence, TF binding sites, splice sites and RNA editing sites but have reduced roles in transcriptional or epigenetic regulation. Furthermore, we use a Drosophila cell culture system and transgenic Drosophila embryos to validate the notion of UCE combinatorial regulatory roles using an eUCE within the *Hox* gene *Ultrabithorax* and show that its protein-coding region also contains alternative splicing regulatory information. Taken together our experiments indicate that UCEs emerge as a result of combinatorial gene regulatory roles and highlight common features in mammalian and insect UCEs implying that similar processes might underlie ultraconservation in diverse animal taxa.

**KEYWORDS**

Ultraconserved elements; UCEs; alternative splicing; epigenetic regulation; transcriptional regulation; *Hox* genes; organismal development.

**BACKGROUND**

Evolutionary conservation of genomic sequences is highly heterogeneous: poorly conserved regions are commonly intermingled with sequences that show perfect conservation across large evolutionary distances. This latter category includes the remarkable class of ultraconserved elements (UCEs), originally defined as sequences of at least 200 nt that are identical across the human, mouse and rat genomes ([Bejerano et al, 2004](#_ENREF_7)). In spite of over a decade of research on UCEs and their detection in vertebrates, insects and other animals, as well as yeasts and plants ([Kellis et al, 2003](#_ENREF_36); [Glazov et al, 2005](#_ENREF_29); [Siepel et al, 2005](#_ENREF_72); [Kritsas et al, 2012](#_ENREF_40); [Reneker et al, 2012](#_ENREF_65); [Ryu et al, 2012](#_ENREF_69)), there is no unifying molecular mechanism that satisfactorily explains their extreme evolutionary conservation ([Harmston et al, 2013](#_ENREF_32)).

Mammalian UCEs have been linked to a diverse set of regulatory functions including transcriptional enhancers ([Woolfe et al, 2005](#_ENREF_80); [Pennacchio et al, 2006](#_ENREF_59); [Lampe et al, 2008](#_ENREF_41); [Visel et al, 2008](#_ENREF_78); [Viturawong et al, 2013](#_ENREF_79)) and non-coding RNAs (ncRNAs) ([Feng et al, 2006](#_ENREF_28); [Calin et al, 2007](#_ENREF_15); [Mestdagh et al, 2010](#_ENREF_53); [Berghoff et al, 2013](#_ENREF_8); [Liz et al, 2014](#_ENREF_47); [Nielsen et al, 2014](#_ENREF_56)). UCEs that overlap with protein-coding genes have also been implicated in RNA regulatory processes, such as alternative splicing, nonsense-mediated RNA decay and RNA editing ([Bejerano et al, 2004](#_ENREF_7); [Siepel et al, 2005](#_ENREF_72); [Lareau et al, 2007](#_ENREF_43); [Ni et al, 2007](#_ENREF_55)). Nonetheless, given that none of these mechanisms seems sufficient to explain the phenomenon of ultraconservation on its own, it has been proposed that superimposed functional constraints might contribute to the generation of UCEs ([Siepel et al, 2005](#_ENREF_72); [Lampe et al, 2008](#_ENREF_41); [Viturawong et al, 2013](#_ENREF_79)).

The genomes of *D. melanogaster* and related species harbour an independent set of UCEs to those found in mammals ([Glazov et al, 2005](#_ENREF_29)). These sequences are shorter than UCEs shared across equally divergent vertebrate genomes ([Makunin et al, 2013](#_ENREF_50)), but were shown to experience a higher degree of selective constraint than mammalian UCEs ([Kern et al, 2015](#_ENREF_38)).

Despite the independent evolutionary origin of mammalian and Drosophila UCEs, their comparison reveals a number of common and distinct features. In regards to the latter, while in mammals it has been difficult to link mutations in UCEs to phenotypic outcomes ([Drake et al, 2006](#_ENREF_24); [Ahituv et al, 2007](#_ENREF_2); [Chen et al, 2007](#_ENREF_18); [Yang et al, 2008](#_ENREF_81); [Catucci et al, 2009](#_ENREF_16); [Poitras et al, 2010](#_ENREF_60); [Chiang et al, 2012](#_ENREF_19)), a recent survey of eleven insertions into Drosophila UCEs identified four as recessive lethal ([Makunin et al, 2013](#_ENREF_50)), illustrating a more straightforward association between UCEs and phenotypes in the fly, making Drosophila a promising model system to study the mechanisms that lead to ultraconservation. As for similarities, fly and mammalian UCEs tend to cluster around genes involved in developmental processes ([Bejerano et al, 2004](#_ENREF_7); [Boffelli et al, 2004](#_ENREF_11); [Sandelin et al, 2004](#_ENREF_70); [Glazov et al, 2005](#_ENREF_29)) and seem associated to related regulatory mechanisms including alternative splicing and RNA editing ([Glazov et al, 2005](#_ENREF_29); [Glazov et al, 2006](#_ENREF_30); [Kern et al, 2015](#_ENREF_38)); however, to date, Drosophila UCEs were not found to overlap with transcription factor binding sites ([Glazov et al, 2005](#_ENREF_29)). A deeper understanding of Drosophila UCEs combined with the use of the powerful genetic tools available in the fruitfly might therefore reveal general and fundamental properties of ultraconservation and its links to the genetic programs encrypted in the genome.

Here we investigate the functional roles of 1,516 UCEs shared across twelve Drosophila genomes. In contrast to previous findings we find that intronic and intergenic Drosophila UCEs (iUCEs) are indeed overrepresented within annotated regulatory elements and show significant enrichments and depletions for binding of specific transcription factors. Our analysis of individual DNA-binding factors also shows that UCEs are enriched for Polycomb-group (PcG) protein binding, suggesting a role for UCEs in chromatin regulation during development. We further show that exonic UCEs (eUCEs) are strongly enriched for multifunctional sequences and are about 100-fold more likely to combine protein-coding capacity with the presence of RNA editing sites, splicing regulators and TF binding sites compared to randomly chosen exonic elements of identical size. To explore whether such predicted multi-functionality was relevant to gene expression we studied one of the Drosophila genes bearing the highest number of UCEs – the *Hox* gene *Ultrabithorax* (*Ubx*) – and observed that mutation of one ultraconserved exon of *Ubx* affects alternative splicing in Drosophila cells in culture and during embryonic development.

Our work therefore contributes to the understanding of the mechanisms that lead to the existence of UCEs and suggests that constraints derived from their multiple enrolment in diverse gene regulatory processes can explain the high level of evolutionary conservation observed in UCEs.

**RESULTS**

***A novel approach for UCE identification finds more than 1,500 UCEs shared across twelve Drosophila genomes***

In this study we define UCEs as DNA elements of at least 50 nt that are identical across the genomes of twelve Drosophila species. Our design is such that the evolutionary distance between the most divergent fruitfly species in our dataset exceeds that of humans and reptiles ([Stark et al, 2007](#_ENREF_73); [Makunin et al, 2013](#_ENREF_50)). Although the original criteria for UCE annotation relied on fewer species ([Bejerano et al, 2004](#_ENREF_7); [Glazov et al, 2005](#_ENREF_29)), we reasoned that the inclusion of more species would be especially valuable for the identification and analysis of eUCEs which might overlap with coding sequences under strong purifying selection. To identify the full set of Drosophila UCEs, we extracted all unique 50-mers from the *D. melanogaster* genome and checked for their presence in the other eleven genomes using the short read mapper Bowtie ([Langmead et al, 2009](#_ENREF_42)). All universal 50-mers were then extracted and, where appropriate, reassembled into longer UCEs (see Materials and Methods). In this manner, we identified a total of 1,516 Drosophila UCEs (Figure 1; Supplementary Table 1).

We assessed the accuracy of our method by manually inspecting each of the 466 UCEs detected on the *D. melanogaster* chromosome arm 3R (31% of our total dataset) in the 15-way multiple alignment available from the dm3 release of the UCSC Genome Browser (<https://genome.ucsc.edu/>) (Methods and Materials; Supplementary Table 1). We found that 345 out of the 466 UCEs (74.0%) were intact in the alignment, *i.e.*, aligned across the 12 *Drosophila* species without mismatches or gaps (Fig. 1D). A further 115 UCEs (24.7%) could be recovered after taking into account different types of alignment ambiguities and errors, which might have prevented their detection using an alignment-based approach: 86 were disrupted due to the insertion of a gap at an ambiguous position, 19 had been split across alignment blocks although the sequence was contiguous in all 12 species and 10 appeared non-conserved due to more extensive alignment or assembly errors (Supplementary Table 1). A total of 6 UCEs (1.3%) could not be verified to be present in a syntenic location in all genomes, typically because they occurred in multiple copies, due to duplications and/or assembly errors. Thus, our alignment-free method greatly increases the sensitivity of UCE detection in these 12 *Drosophila* genomes, at the cost of a small false positive rate in the order of 1%. As a case in point, a recent study of the same 12 species identified 98 UCEs of at least 80 nt based on the multiple alignment used above ([Kern et al, 2015](#_ENREF_38)), whereas our method identified 131 such UCEs, an increase of 34%.

Notably, the UCEs discovered were not randomly distributed: they occurred in clusters within the *D. melanogaster* genome (observed median distance between UCEs: 18 kb; expected: 56 kb; p < 10^-15^, Mann-Whitney test). The largest UCE clusters overlapped with key developmental factors, including the pair-rule gene *odd-skipped* (*odd*), the *Hox* genes *Antennapedia* (*Antp*) and *Ultrabithorax* (*Ubx*) (see below) and the *Hox* co-factor *homothorax* (*hth*) (Table 1; Materials and methods). The association between UCEs and developmental genes was further confirmed by gene ontology (GO) analysis ([Ashburner et al, 2000](#_ENREF_5); [Eden et al, 2009](#_ENREF_26)) which revealed significant enrichments of GO categories such as “organ development”, “pattern specification process” and “regulation of transcription from RNA polymerase II promoter” among the most UCE-rich genes (Supplementary Table 2; Materials and methods). Thus our novel approach applied to distantly related Drosophila species points towards a general and robust association between ultraconservation and the genetic control of animal development, in line with earlier findings regarding Drosophila and mammalian UCEs ([Sandelin et al, 2004](#_ENREF_70); [Glazov et al, 2005](#_ENREF_29); [Kern et al, 2015](#_ENREF_38)).

***UCEs are selectively enriched for transcriptional regulators in early development***

The detection of multiple links between Drosophila UCEs and developmental genes and processes (see above) together with the fact that in mammals many UCEs serve as developmental enhancers ([de la Calle-Mustienes et al, 2005](#_ENREF_21); [Woolfe et al, 2005](#_ENREF_80); [Pennacchio et al, 2006](#_ENREF_59); [Visel et al, 2008](#_ENREF_78)) prompted us to consider a plausible role of Drosophila UCEs in transcriptional regulation.

In contrast to the findings of an earlier study ([Glazov et al, 2005](#_ENREF_29)) we found an enrichment of Drosophila UCEs in annotated regulatory regions: we detected 21 UCEs that overlapped with known regulators in the ORegAnno database ([Griffith et al, 2008](#_ENREF_31)), which represented a 1.7-fold enrichment compared to randomly chosen genomic elements (p = 0.015,  *χ^2^* test). Motivated by this finding, which suggests a functional parallel between mammalian and Drosophila UCEs, we decided to undertake a more detailed investigation of the contributions of UCEs to transcriptional regulation in Drosophila.

For this we intersected our UCE annotations with data from the modENCODE consortium ([Celniker et al, 2009](#_ENREF_17)) providing the binding sites of 34 TFs in early development (see Materials and Methods). UCEs that overlapped with annotated ncRNAs were excluded from this analysis, since many ncRNAs are highly expressed and therefore prone to give rise to false positives in chromatin immunoprecipitation (ChIP) experiments ([Teytelman et al, 2013](#_ENREF_76)). We assessed each TF for enrichment or depletion within UCEs compared to reference elements and detected several cases where patterns of TF binding differed significantly between UCEs and reference elements (Fig. 2A) suggesting that ultraconservation is associated with specific regulatory networks. The list of significantly enriched TFs included developmental factors such as Hairy, which plays a critical role in the segmentation of the early embryo ([Nusslein-Volhard & Wieschaus, 1980](#_ENREF_57)), Fruitless, which promotes male-specific neural development and behaviour ([Manoli et al, 2005](#_ENREF_51)), as well as Homothorax, a Hox protein co-factor ([Ryoo et al, 1999](#_ENREF_68)).

We further reasoned that, if the enrichment of TFs within UCEs is biologically relevant in the context of Drosophila development, we might observe differential TF enrichment at various developmental time points. For one of the enriched TFs, Caudal (Cad) ([Mlodzik et al, 1985](#_ENREF_54)), data were available for different developmental stages enabling us to use this protein to explore the ways in which TF binding relates to UCE function at different developmental time points. This analysis showed that in young embryos Cad binding was significantly enriched in iUCEs (intronic), while in adult flies there was a depletion of Cad binding in this type of UCE (Fig. 2C). This observation is consistent with a role for UCEs in the dynamic transcriptional processes that control development. Taken together, our results indicate that many Drosophila UCEs, especially iUCEs, act as enhancers in particular during early Drosophila development.

***UCEs are bound by Polycomb-group proteins***

The protein Polycomblike (Pcl) was one of the most consistently enriched TFs in our analysis (Fig. 2A). Because Pcl binds to Polycomb response elements (PREs) ([Papp & Muller, 2006](#_ENREF_58)), we speculated that UCEs might be associated with Polycomb-group (PcG) proteins, which mediate epigenetic silencing of gene expression and target many genes with critical roles in development including the *Hox* genes ([Steffen & Ringrose, 2014](#_ENREF_74)). To examine this possibility we looked at the binding patterns of Polycomb (Pc), Posterior sex combs (Psc) and Sex combs extra (Sce), which form part of the Polycomb repressive complex 1 (PRC1), Enhancer of zeste (E(z)), which forms part of Polycomb repressive complex 2 (PRC2), as well as the Trithorax-group protein Absent, small, or homeotic discs 1 (Ash1), which is associated with non-repressed PcG targets ([Steffen & Ringrose, 2014](#_ENREF_74)). All four PcG proteins were significantly enriched at iUCEs (Fig. 2B). Pc and E(z) were also enriched at eUCEs, while Psc and Sce were depleted from these sequences, suggesting potentially divergent mechanisms of PcG protein function at different types of UCEs. In contrast, Ash1 was significantly depleted from all UCE classes, consistent with the antagonistic relationship between PcG and TrxG proteins. These results add further context to the genomic association between UCEs and developmental regulators and suggest that Drosophila UCEs might be implicated in the establishment and maintenance of chromatin states necessary for precise temporal control of gene expression throughout development.

***Multifunctional sequences are strongly enriched among eUCEs***

Due to their involvement in protein encoding eUCEs are likely to be subjected to distinct functional requirements from those found in iUCEs. Apart from constraints on coding sequences, previous work highlighted RNA editing and splicing as two individual processes associated with eUCEs in flies and mammals ([Bejerano et al, 2004](#_ENREF_7); [Glazov et al, 2005](#_ENREF_29); [Lareau et al, 2007](#_ENREF_43); [Ni et al, 2007](#_ENREF_55)), but their cumulative contribution had not been considered. We hypothesised that combinations of several molecular functions within a single sequence might collectively constrain sequence evolution and explain the presence of eUCEs in Drosophila.

To test whether this was the case we calculated the degree of multifunctionality observed for UCEs and reference elements by assessing each UCE in terms of protein-coding potential, presence of RNA editing sites ([Ramaswami & Li, 2014](#_ENREF_63)), overlap with intron-exon boundaries and presence of TF binding sites ([Celniker et al, 2009](#_ENREF_17)). We observed a clear shift in the UCE distribution towards a larger number of molecular functions per element (Fig. 3A; p = 1.9 x 10^-8^, Mann-Whitney test) suggesting that multifunctionality is a core property of UCEs. As an example, our analysis showed that eUCEs are approximately 100-fold more likely than reference elements to be tetrafunctional, *e.g.*, they are located in a protein-coding region, overlap with a splice site, contain an RNA editing site and are bound by at least one TF (Fig. 3B, Supplementary Table 3).

Notably, our analysis of eUCEs also revealed that almost all (96%) eUCEs overlap with alternatively spliced exons (expectation based on reference elements: 47%; p = 6.8 x 10^-12^, *χ^2^*-test) suggesting an important role for ultraconservation in the generation of alternative transcripts. This observation prompted us to consider specific eUCEs to experimentally test the hypothesis that ultraconservation might be related to multifunctional roles played by UCEs (see below).

***Multifunctionality underlies ultraconservation of an alternatively spliced exon in the* Hox *gene* Ubx**

One of the eUCEs in our dataset overlaps with a small exon (51 nt) in the Drosophila *Hox* gene *Ubx* (Fig. 4A). Given that the *Hox* genes represent a gene class characterised for its high number of eUCEs in mammals ([Lampe et al, 2008](#_ENREF_41); [Lin et al, 2008](#_ENREF_46)) we decided to investigate the *Ubx* eUCE in higher detail. Alternative splicing of this *Ubx* exon, known as microexon I (mI), as well as of an additional small exon, microexon II (mII), generates functionally distinct *Ubx* isoforms ([Reed et al, 2010](#_ENREF_64); [de Navas et al, 2011](#_ENREF_22)). The demonstrated molecular and developmental relevance of mI alternative splicing together with the evolutionary conservation of *Ubx* splicing patterns and motifs across distantly related Drosophila species ([Bomze & Lopez, 1994](#_ENREF_12); [Hatton et al, 1998](#_ENREF_33)) brought us to hypothesise that mI ultraconservation might be a consequence of overlapping constraints derived from the necessity of maintaining both a particular protein-coding sequence and specific splicing regulatory elements within mI.

To evaluate this hypothesis, we first investigated to what extent the mI protein-coding sequence showed signs of purifying selection. Using BLAST ([Altschul et al, 1990](#_ENREF_3)), we were able to locate the mI sequence in the genomes of three outgroup species to the Drosophila clade: the common housefly *Musca domestica*, the tsetse fly *Glossina morsitans* and the Mediterranean fruit fly *Ceratitis capitata* (Materials and methods). We then estimated the rate of nonsynonymous substitutions relative to the rate of synonymous substitutions (dN/dS) with codeml ([Yang, 1997](#_ENREF_82)). The dN/dS value was 0.045, consistent with strong purifying selection acting on the mI coding sequence. The conservation of mI at nonsynonymous sites can therefore be fully or partly explained by coding constraints. Next, we tested whether the conservation at synonymous sites was due to selective constraints or a consequence of insufficient divergence times between species, which might not have allowed mutations at synonymous sites to occur. To this end, we compared the mI exon to the *Ubx* homeodomain, a sequence encoding 60 amino acids (aa) identical across all twelve investigated Drosophila species. Focusing on the third position of each codon, we found that 29 out of 57 sites within the homeodomain had synonymous substitutions. Using this as our reference we were thus able to exclude that the lack of synonymous mutations at the 15 synonymous sites within the mI exon was due to chance (p = 0.0002, Fisher’s exact test) supporting our hypothesis that coding constraints contribute to, but cannot fully explain, ultraconservation within the mI exon.

Building on these observations we decided to use the UCE in *Ubx* mI (mI-UCE) to test the possibility that it functions as a protein-coding element as well as a docking region for splicing factors. We reasoned that if the latter were true, an experiment where coding sequence capacity is maintained while the nucleotide sequence is modified should potentially expose such splicing-related functions. To test this model, we decided to carry out a series of experiments where the original sequence of the mI-UCE was replaced by one in which the coding potential was unaffected but the nucleotide composition distorted (Fig. 4B). Both versions of the mI-UCE (wild type and mutated (mutA)) were subcloned within a *Ubx* splicing minigene previously shown to produce a relatively complex pattern of alternatively spliced products ([Hatton et al, 1998](#_ENREF_33)). We then proceeded to test the alternative splicing patterns that resulted from these constructs in Drosophila S2 cells in culture. Here we observed that the expression level of mI-bearing mRNAs was significantly reduced in the presence of the mI mutation (Fig. 4C). These results together with the fact that overall expression levels of *Ubx* mRNAs do not differ across the genotypes suggest that mI mutation indeed affects *Ubx* alternative splicing in Drosophila cells.

To explore the extent to which our observations in cultured cells were also valid in the physiological context of the developing fruitfly embryo, we created a series of transgenic Drosophila lines carrying wild type and mutA versions of mI-UCE in *Ubx* minigenes whose expression could be controlled via the Drosophila UAS/Gal4 system ([Brand & Perrimon, 1993](#_ENREF_13)). For our analysis we chose to activate the expression of the *Ubx* minigenes within the embryonic central nervous system (CNS) given that the splicing patterns of *Ubx* are complex within this tissue ([Lopez & Hogness, 1991](#_ENREF_48); [Artero et al, 1992](#_ENREF_4); [Reed et al, 2010](#_ENREF_64); [Thomsen et al, 2010](#_ENREF_77)). Remarkably, the results of these experiments in developing embryos (Fig. 4D-E) nicely match those observed in cultured cells: lower levels of mI-bearing mature mRNAs were detected in the presence of the mI mutation.

Furthermore, the biological significance of the observed changes in the pattern of Ubx alternative splicing generated via UCE mutation is evident given that Ubx splicing isoforms have been shown to: (i) have different abilities to bind to DNA targets *in vitro* ([Reed et al, 2010](#_ENREF_64)), (ii) display isoform–specific gene activation patterns *in vivo* in two developmental contexts: within the developing embryo i.e. regulation of both: the *decapentaplegic* (*dpp*) promoter and endogenous gene, and during the formation of adult appendages – i.e. regulation of *wingless, araucan* and *spalt* genes in wing and haltere imaginal discs ([Reed et al, 2010](#_ENREF_64); [de Navas et al, 2011](#_ENREF_22)), (iii) perform different roles during the development of the peripheral nervous system (PNS) in the embryo ([Reed et al, 2010](#_ENREF_64)) and during haltere development in the adult ([de Navas et al, 2011](#_ENREF_22)) and (iv) induce different patterns of neural differentiation during the formation of the embryonic nervous system ([Rogulja-Ortmann et al, 2014](#_ENREF_67)).

All in all, the data of our experimental analysis in cultured cells and *in vivo* support a model by which the mI-UCE performs functions that affect the process of alternative splicing; these data support our hypothesis that UCEs may have retained their sequences over long evolutionary periods due to their intrinsic multifunctionality in regards to gene expression control.

**DISCUSSION**

Despite a decade of research since the discovery of UCEs the molecular basis of ultraconservation remains unknown. So far, most UCE studies have been carried out in mammals, yet Drosophila UCEs are currently receiving renewed attention ([Makunin et al, 2013](#_ENREF_50); [Makunin et al, 2014](#_ENREF_49); [Kern et al, 2015](#_ENREF_38)), not least because of the clear phenotypic effects that can be observed in UCE mutants in fruitflies ([Makunin et al, 2013](#_ENREF_50)). Nevertheless, the molecular functions of Drosophila UCEs have not been extensively investigated, leaving open the question of whether similar molecular mechanisms underlie ultraconservation in insects and mammals. Here we combine a computational approach with molecular experiments in Drosophila cells and embryos and gene regulatory data from the modENCODE consortium ([Celniker et al, 2009](#_ENREF_17)) to provide a modern and comprehensive functional overview of more than 1,500 UCEs shared across twelve Drosophila genomes.

Previous work suggested that Drosophila UCEs are not involved in transcriptional regulation ([Glazov et al, 2005](#_ENREF_29); [Kern et al, 2015](#_ENREF_38)). In contrast, our analysis of the binding sites of 34 TFs revealed that many iUCEs likely serve as enhancers in early fly development, similarly to what has been observed in mammals ([Pennacchio et al, 2006](#_ENREF_59); [Visel et al, 2008](#_ENREF_78)). Furthermore, at least in some cases, UCE enhancer activity appears to be temporally restricted as suggested by our observations of Cad binding at different developmental time points. We also observed significant enrichment of PcG proteins at UCEs, thus extending previous findings of high conservation at individual PREs ([Dellino et al, 2002](#_ENREF_23)) and indicating that UCEs might be involved in epigenetic silencing during development. These observations also suggest that –in evolutionary terms– chromatin silencing might be a particularly well-preserved function. These data might also hint that the roles of UCEs in epigenetic regulation differ between Drosophila and mammals given the previously observed depletion of PcG proteins at UCEs in mouse embryonic stem cells ([Viturawong et al, 2013](#_ENREF_79)); alternatively, these seemingly distinct results might reflect the dynamic nature of PcG-mediated silencing, especially given that another study reported an association between PcG proteins and highly conserved mammalian sequences ([Lee et al, 2006](#_ENREF_44)). Taken together, our analysis of the binding sequences of TFs and Polycomb proteins support an integral role for iUCEs in developmental gene regulation.

In contrast, eUCEs show less pronounced patterns of TF binding enrichment and depletion, suggesting that they do not primarily function as transcriptional regulators. Indeed, eUCEs have been associated with other regulatory processes, such as alternative splicing and RNA editing ([Glazov et al, 2005](#_ENREF_29); [Kern et al, 2015](#_ENREF_38)). In this regard, our data shows a statistically significant association between eUCEs and alternatively spliced exons. Although we sought to determine whether UCEs had a distinctive link with splicing regulatory elements the size of our eUCE dataset was too small to probe this possibility using computational methods (Materials and methods).

Furthermore, our analysis shows that multiple functional layers are frequently superimposed on a single eUCE sequence. For example, we show that eUCEs are nearly 100-fold more likely than expected to simultaneously contain protein-coding sequence, TF binding sites, splice sites and RNA editing sites. This finding adds to the growing literature on regulatory sequences embedded within coding regions ([Lin et al, 2011](#_ENREF_45); [Stergachis et al, 2013](#_ENREF_75); [Birnbaum et al, 2014](#_ENREF_9)) and suggests that many eUCEs represent extreme cases of “genomic multitasking”.

We experimentally evaluated one such multitasking element, an eUCE overlapping the short mI exon within the *Hox* gene *Ubx.* Although the protein-coding sequence is under strong purifying selection, our comparison between the mI sequence and that of the well-conserved homeodomain showed that protein-coding constraints alone were insufficient to explain the ultraconservation of this exon. As the mI exon is alternatively spliced, we used a previously developed splicing minigene system ([Hatton et al, 1998](#_ENREF_33)) to analyse splicing patterns of the wild type *Ubx* gene and a version of *Ubx* where the mI exon contained synonymous substitutions. The results of these experiments showed clear differences in *Ubx* splicing, both in cell culture and fly embryos, demonstrating that the mI exon carries out two functions in parallel: it encodes an evolutionarily conserved protein sequence and regulates its own splicing. Interestingly, many alternatively spliced short exons overlap with UCEs also in mammalian genomes ([Bejerano et al, 2004](#_ENREF_7)), suggesting that our findings might be relevant to the study of UCEs in other animal groups.

The work presented above shows several common features shared between UCEs in mammals and Drosophila. An intriguing possibility that emerges from this work is that these similarities reflect common mechanisms underlying ultraconservation in distantly related animals. In addition, our findings support the hypothesis that UCEs are sculpted by overlapping functional constraints, in particular for eUCEs, and suggest that further functional dissection of Drosophila UCEs will lead to general insights into the selective forces that shape gene regulatory elements in animal genomes.

In summary, we have performed a functional survey of 1,516 UCEs that are shared across twelve Drosophila genomes. Our findings support a role for iUCEs in the transcriptional and epigenetic regulation of genes involved in early fly development. In addition, we found that eUCEs are shaped by cumulative functional constraints and are two orders of magnitude more likely than expected to contain protein-coding sequence, transcription factor binding sites, RNA editing sites and splice sites within a single element. We experimentally characterised an eUCE found in the *Hox* gene *Ultrabithorax* and showed that the extreme conservation of this element is due to both protein-coding constraints and the presence of splicing regulators that modulate the balance of biologically distinct *Ubx* isoforms in cell culture and developing fly embryos. Our results highlight similarities between UCEs in Drosophila and mammals, pointing to a shared molecular mechanism underlying these independently evolved elements.

**MATERIALS AND METHODS**

***Identification of ultraconserved elements in twelve genomes***

Genome assemblies for *D. ananassae* (droAna3), *D. erecta* (droEre2), *D. grimshawi* (droGri2), *D. melanogaster* (dm3), *D. mojavensis* (droMoj3), *D. persimilis* (droPer1), *D. pseudoobscura* (dp4), *D. sechellia* (droSec1), *D. simulans* (droSim1), *D. virilis* (droVir3), *D. willistoni* (droWil1) and *D. yakuba* (droYak2) were downloaded from the UCSC Genome Browser ([Adams et al, 2000](#_ENREF_1); [Kent et al, 2002](#_ENREF_37); [Richards et al, 2005](#_ENREF_66); [Drosophila 12 Genomes Consortium, 2007](#_ENREF_25)). We extracted all 50-mers that occurred a single time in the *D. melanogaster* genome and mapped them to the other eleven genomes using Bowtie release 0.12.7 ([Langmead et al, 2009](#_ENREF_42)). Only 50-mers with perfect matches (bowtie -v 0) in all genomes were kept for further analysis. To retrieve full-size ultraconserved elements, we fused overlapping 50-mers into longer sequences and verified that the reconstituted elements were found in all genomes.

Earlier studies of *Drosophila* UCEs have defined ultraconservation using different criteria ([Glazov et al, 2005](#_ENREF_29); [Makunin et al, 2013](#_ENREF_50); [Kern et al, 2015](#_ENREF_38)). We chose a cutoff of 50 nt to be consistent with the original study by Glazov et al ([2005](#_ENREF_29)) that focussed on UCEs in the *D. melanogaster* and *D. pseudoobscura* genomes. Based on the overall similarity of these two genomes, [Glazov et al (2005)](#_ENREF_29) estimated the false positive rate in their study to be only 0.4%. Given that our analysis included ten additional species, of which three are more distantly related, the proportion of UCEs in our dataset that are explained by overall sequence similarity should be substantially lower than 0.4% and can therefore be considered negligible.

Unlike previous approaches, our method does not rely on whole-genome alignments and does not incorporate information on synteny. To assess whether our dataset included UCEs that did not occur at syntenic positions across the 12 genomes, we carefully inspected the 466 UCEs located on the *D. melanogaster* chromosome arm 3R in the Multiz 15-way whole-genome alignment available from the dm3 release of the UCSC Genome Browser (https://genome.ucsc.edu/), which was previously used by [Kern et al (2015)](#_ENREF_38). We first divided UCEs into four groups: 1) perfect correspondence between our annotation and the alignment, 2) perfect correspondence after the position of an ambiguously placed gap had been adjusted, 3) perfect correspondence, but due to an outgroup species the alignment spanned two or more alignment blocks and 4) incomplete correspondence. For the UCEs in the fourth group, we assessed whether our annotated UCE occurred in a syntenic position, which we defined as the region between the two UCEs that neighboured the UCE in *D. melanogaster.* Instances where we did not find our annotated UCE in a syntenic position, and where this could not be explained by alignment or assembly errors, were due to multiple occurrences of the UCE and highly similar sequences in one or more of the non-melanogaster genomes. In these cases, the annotated UCE in *D. melanogaster* had been aligned to a UCE-like sequence, which typically contained only a single mismatch (Supplementary Table 1).

***Annotation of UCE clusters***

We grouped UCEs if they occurred within less than the median distance (18 kb) of each other. This resulted in 288 UCE clusters, comprising 1043 UCEs (Supplementary table 4). To explore the association between UCEs and protein-coding genes, we chose to focus on the 15 largest clusters (each comprising at least 10 UCEs), as the limited quality of some of the analysed genomes prevented us from performing a global clustering and synteny analysis for all species. For these clusters, we checked the overlap with protein-coding genes in the *D. melanogaster, D. pseudoobscura* and *D. virilis* genomes (Table 1).

***Comparisons of UCEs and genomic reference elements***

We created a reference dataset by dividing the *D. melanogaster* genome into fragments with the same length distribution as the UCE set. All reference elements were required to map uniquely to the genome (bowtie -v 0 -m 1). The reference elements were grouped into functional classes (intergenic, intronic, exonic or ncRNA) in the same manner as the UCEs. We used BEDTools ([Quinlan & Hall, 2010](#_ENREF_61)) to define the overlap between UCEs or reference elements with various genomic annotations, and carried out the statistical analyses with R version 2.12.2 ([R Development Core Team, 2011](#_ENREF_62)).

***Gene ontology analysis***

We searched for GO categories that were associated with the most UCE-rich genes using the GOrilla tool ([Ashburner et al, 2000](#_ENREF_5); [Eden et al, 2009](#_ENREF_26)), which identifies enriched GO terms for ranked gene lists. We ranked genes based on the number of UCEs within each gene, including flanking regions of 10 kb upstream and downstream, divided by the number of reference elements in the same interval.

***Analysis of modENCODE data***

We analysed ChIP-chip and ChIP-seq data from the modENCODE consortium ([Celniker et al, 2009](#_ENREF_17)). TFs were chosen based on association with the GO term GO:0003700 (sequence-specific DNA binding transcription factor activity) ([Ashburner et al, 2000](#_ENREF_5)) and the availability of data from embryos younger than twelve hours. PcG and TrxG proteins were chosen based on annotations provided by Steffen and Ringrose ([2014](#_ENREF_74)). For the PcG/TrxG analysis, we did not limit our analysis to a specific developmental stage, but merged all available datasets for each protein. A list of the precise datasets we used is included in Supplementary Table 5. We tested for enrichment or depletion of each factor within intergenic, intronic (iUCEs) or exonic UCEs (eUCEs) relative to reference elements (see above) using a *χ^2^*-test. Correction for 117 multiple tests was performed using the Benjamini-Hochberg method.

***Global analysis of alternative splicing***

To evaluate the overlap between eUCEs and alternatively spliced exons, we downloaded *D. melanogaster* coordinates for constitutive and non-constitutive exons from Ensembl release 79 ([Cunningham et al, 2015](#_ENREF_20)) and intersected these with our set of eUCEs and exonic reference elements using BEDTools ([Quinlan & Hall, 2010](#_ENREF_61)).

We obtained sequences of 99 putative exonic splicing enhancers (ESEs) from Brooks et al ([2011](#_ENREF_14)). Fore each motif, we counted the number of matching eUCEs and exonic reference elements (see above) and evaluated the statistical significance a *χ^2^*-test. Correction for 99 multiple tests was performed using the Benjamini-Hochberg method. In addition, we used DREME ([Bailey, 2011](#_ENREF_6)) with default settings to search for *de novo* motifs within the eUCEs compared to shuffled sequences.

***Phylogenetic analysis of the mI exon***

We used the mI aa sequence in a tblastn BLAST search ([Altschul et al, 1990](#_ENREF_3)) against the NCBI nucleotide collection (http://blast.ncbi.nlm.nih.gov) and recovered mI in the common housefly *Musca domestica* ([Scott et al, 2014](#_ENREF_71)) and the Mediterranean fruit fly *Ceratitis capitata* (http://www.hgsc.bcm.edu), but not in more distantly related species, such as the mosquitoes *Anopheles gambiae* and *Aedes aegypti*, the flour beetle *Tribolium castaneum* or the honey bee *Apis mellifera.* We used the same method to identify the mI exon in the genome of the tsetse fly *Glossina morsitans* ([International Glossina Genome Initiative, 2014](#_ENREF_35))*,* made available through VectorBase ([Megy et al, 2012](#_ENREF_52)). To generate an alignment of the surrounding introns, we extended the sequences by including 50 nt on each side of the mI exon and aligned the sequences with MUSCLE ([Edgar, 2004](#_ENREF_27)). For the coding part of the alignment, we estimated dN/dS for the whole tree using codeml and standard settings ([Yang, 1997](#_ENREF_82)).

***Fly stocks and embryo collections***

Virgins of transgenic UAS-lines were crossed to males of 69B-Gal4 (Bloomington 1774) or *elav*-Gal4 (a gift of Matthias Soller, Birmingham, United Kingdom) fly lines. Embryos were collected at 25C in the dark on apple juice plates supplemented with yeast paste following standard procedures.

***Generation of Ubx-mutA constructs***

The Ubx.4 plasmid, which carries a *Ubx* wild type minigene, was originally developed in the laboratory of Javier Lopez ([Hatton et al, 1998](#_ENREF_33)). We introduced synonymous mutations into the mI exon (Fig. 4B) by PCR-driven overlap extension ([Heckman & Pease, 2007](#_ENREF_34)). The PCR fragment was cloned into the pGEM-T Easy vector (Promega), which was sequentially digested with AflII and PmlI to release a 255 nt fragment. The fragment was then cloned into the Ubx.4 plasmid to generate the derivative construct Ubx.mutA.

***Subcloning and transgenesis***

To create UAS-Ubx minigene expression constructs bearing wild type (wt) or mutated versions of the mI microexon (mutA) the following procedures were employed. For the creation of pUAS.Ubx.mini.HA.short.attB (HA.short) the *Ubx* wild type minigene was released from Ubx.4 via a partial NruI digest followed by a SacII digestion; the resulting 6241 nt fragment was cloned into pBluescript to form pBSII.SK(+).Ubx.mini.HA.short. Subsequently, the minigene was released by sequential SpeI/blunting through T4 Polymerase/KpnI digestions and transferred to the transformation vector pUASP.K10.attB (a gift from Beat Suter, Bern, Switzerland) ([Koch et al, 2009](#_ENREF_39)) that had been sequentially treated with NdeI/blunting through T4 Polymerase/KpnI. To create the expression construct pUAS.Ubx.mini.HA.short.mI.mutA.attB (HA.short.mI.mutA), a mutant version of mI was transferred from Ubx.4.mutA to pBSII.SK(+).Ubx.mini.HA.short using a NdeI/NsiI digest; the mutant minigene was then transferred to pUASP.K10.attB as described above. All enzymes were from *NEB*. Injection of UASP.attB constructs as well as the screening for and balancing of transformants was performed by BestGENE (<http://www.thebestgene.com/>) using the ZH-attB-51C landing site ([Bischof et al, 2007](#_ENREF_10)).

***Antibody labelling***

At the protein level *Ubx*-mini transgene expression was detected by antibody-stains using enzymatic, alkaline phosphatase detection using standard protocols. In brief, we used anti-hemagglutinin (HA) (Covance; 1:400) followed by anti-mouse-biotin (Sigma-Aldrich,1:200) and streptavidin-alkaline phosphatase conjugates (Roche, 1:5000); enzymatic detection was performed using NBT/BCIP (Roche) substrate.

***S2 cell experiments***

Drosophila S2 cells were transfected using Effectene transfection reagent (Qiagen, Valencia, CA) according to the manufacturer's protocol. Typically, 1.5 million cells were transfected with 200ng DNA (10ng and 40ng of Plasmid-minigene, Ubx-wt or mutant, were transfected) and incubated for ~65 hours before collection of RNA.

***RT-PCR analysis***

Total RNA was isolated (Sigma RNA Minikit) followed by DNase treatment. cDNA was generated with oligo-dT primer using either 1 or 3 ug of total RNA with M-MuLV reverse transcriptase from NEB (20ul reaction volume; 1h at 42°C). 1ul of cDNA was used as a template for PCR to amplify isoforms. PCR primers Ubx_E1F: TGGAATGCCAATTGCACCATC3’/Ubx3’R 5’CGCGTCTTCGCAGACCATTT3’ were used to detect the different isoforms (PCR cycles: 31x (94°C 45s, 56°C 1min, 72°C 30s). More PCR cycles amplified other isoforms but gave inconsistent results indicating that the reaction was not in linear range and were not considered further. Minigene expression level was monitored by primers expF: 5’AGTGGAAGGAGCGCAGATTA3’/ expR: 5’TCGAGCGAATCCTCTTGAAT3’ (PCR cycles 25x (94°C 30 s, 56°C 20s, 72°C 20 s)) amplifying a product of 103 nt. Products were separated on an 8% non-denaturing polyacrylamide gel and quantified with MultiGauge (Fujifilm). Background corrected intensity values were normalized to the general gene level and the average and standard error from the three replicas was calculated presented in log2 scale.

**ACKNOWLEDGEMENTS**

We thank Adam Eyre-Walker and Margarida Cardoso Moreira for helpful discussions. This work was funded by an EMBO long-term fellowship to MW and a Wellcome Trust Investigator Award to CRA (098410/Z/12/Z). The authors declare that they have no competing interests. MW and CRA designed the study. MW performed the computational analyses and developed mutated constructs. BH and ST performed experiments in Drosophila cells and embryos. CRA supervised the computational and experimental work. MW and CRA wrote the manuscript.

**TABLES**

**Table 1. The 15 largest UCE clusters in the *D. melanogaster* genome.**

| **Cluster ID** | **Total UCEs** | **Intergenic** | **Intronic** | **Junction** | **Exonic** | **ncRNA** | **Flybase genes^1^** |
| --- | --- | --- | --- | --- | --- | --- | --- |
| cluster_186 | 21 | 15 | 6 | - | - | - | ***Cyp12e1***  ***hth*** |
| cluster_247 | 14 | 14 | - | - | - | - | ***fkh*** |
| cluster_47 | 11 | 11 | - | - | - | - | *CG33648*  ***CG4218*** |
| cluster_147 | 11 | - | 10 | - | 1 | - | ***bru-3*** |
| cluster_180 | 11 | 2 | 7 | - | 2 | - | ***Antp*** |
| cluster_194 | 11 | 10 | 1 | - | - | - | *CG17025* |
| cluster_235 | 11 | - | - | 10 | 1 | - | ***slo*** |
| cluster_253 | 11 | 11 | - | - | - | - | ***CG2267***  *CG31013*  ***PH4alphaPV***  ***CG34432/Spn100A***  *CG34433*  *CG1342*  ***CG12069***  ***CG12066/Pka-C2***  ***CG31010*** |
| cluster_5 | 10 | 6 | 4 | - | - | - | ***CG5397***  ***robo3*** |
| cluster_10 | 10 | 10 | - | - | - | - | ***sob***  ***odd*** |
| cluster_94 | 10 | 10 | - | - | - | - | *CG30447*  *CG10822* |
| cluster_157 | 10 | 10 | - | - | - | - | *CG33259* |
| cluster_187 | 10 | 10 | - | - | - | - | ***hth*** |
| cluster_195 | 10 | 10 | - | - | - | - | ***CG31337***  *CG14370* |
| cluster_208 | 10 | - | 10 | - | - | - | ***Ubx*** |

^1^ Genes in bold are conserved across all twelve investigated Drosophila species and associated with the same cluster in *D. pseudoobscura* and *D. virilis*.

**FIGURE LEGENDS**

**Figure 1. Genomic distribution of Drosophila UCEs. (A)** Relative frequencies of UCEs overlapping ncRNAs, exons, intron-exon junctions, introns and intergenic regions, in comparison to reference elements with the same length distribution drawn from the entire genome. Of the 1,516 UCEs we identified, 186 overlapped with exons of protein-coding genes, 393 were purely intronic, 919 were located in intergenic regions and 18 overlapped with annotated ncRNAs, primarily tRNAs. **(B)** Frequencies of Drosophila UCEs of various lengths. **(C)** Chromosomal location of the Drosophila UCEs in the *D. melanogaster* and *D. pseudoobscura* genomes. For each chromosome, coordinates increase from left to right (*D. melanogaster*) or down to up (*D. pseudoobscura*). UCE clusters with at least ten members are indicated by red circles. **(D)** Validation of the 466 UCEs on *D. melanogaster* chromosome arm 3R in the 15-way alignment available from the dm3 release of the UCSC Genome Browser. The majority of the UCEs from our pipeline were immediately detected in the alignment (“intact UCEs”), while others could be retrieved after taking into account ambiguous gap placements, sequences spanning two or more alignment blocks and other alignment artifacts. See main text for further details.

**Figure 2. Involvement of Drosophila UCEs in transcriptional regulation. (A)** Significant (p < 0.05) enrichment or depletion of 34 transcription factors in UCEs relative to reference elements in early Drosophila development. A *χ^2^*-test was applied to each factor and UCE type, followed by Benjamini-Hochberg correction for multiple tests. The datasets used to generate this figure are listed in Supplementary Table 5. Abbreviations: Bab1, Bric a brac 1; Cad, Caudal; Chinmo, Chronologically inappropriate morphogenesis; Cnc, Cap-n-collar; CTCF, CTCF; D, Dichaete; Disco, Disconnected; Dll, Distal-less; En, Engrailed; Fru, Fruitless; Ftz-f1, Ftz transcription factor 1; GATAe, GATAe; H, Hairy; Hkb, Huckebein; Hr46, Hormone receptor-like in 46; Hth, Homothorax; Inv, Invected; Jumu, Jumeau; Kn, Knot; Kr, Kruppel; Lola, Longitudinals lacking; Pan, Pangolin; Pcl, Polycomblike; Prd, Paired; Run, Runt; Sc, Scute; Sens, Senseless; Sin3A, Sin3A; Stat92E, Signal-transducer and activator of transcription protein at 92E; Su(H), Suppressor of Hairless; Zfh1, Ttk, Tramtrack; Ubx, Ultrabithorax; Usp, Ultraspiracle; Zfh1, Zn finger homeodomain 1. **(B)** Enrichment and depletion of four Polycomb-group and one Trithorax-group proteins. Analyses were performed as in (A). Abbreviations: Ash1, absent, small, or homeotic discs 1; E(z), Enhancer of zeste; Pc, Polycomb; Psc, Posterior sex combs; Sce, Sex combs extra. **(C)** Enrichment and depletion of Cad binding at five points of Drosophila development. Analyses were performed as in (A). Double asterisks indicate 0.01 < p < 0.001 and triple asterisks p < 0.001.

**Figure 3. Overlapping functions of exonic UCEs. A.** Proportion of UCEs and reference elements which overlap with between zero and four types of functional sequences. The investigated functions were protein-coding capacity, splicing (overlap with a splice site), RNA editing (from the RADAR database ([Ramaswami & Li, 2014](#_ENREF_63))) and transcription factor binding (based on modENCODE data ([Celniker et al, 2009](#_ENREF_17))). **B.** Venn diagram of the number of UCEs that overlap with different combinations of functions. The four function types are the same as in B. The number of UCEs in each category is displayed. Colours indicate enrichment or depletion of UCEs relative to the proportion of reference elements that fall within each category.

**­­Figure 4. Analysis of an ultraconserved exon within the *Ubx* gene. (A)** The *Ubx* gene (purple) contains twelve UCEs (red triangles) within the transcribed region. Coding sequences are shown as thick boxes and untranslated regions (UTRs) as narrow boxes. The gene is transcribed from left to right. One of the UCEs overlaps with the alternatively spliced mI exon. An alignment of the mI-UCE sequence from twelve Drosophila species and three more distantly related fly species is shown.

Substitutions relative to the Drosophila sequence are shown in orange and insertions as a vertical line, with the number of inserted bases added above the sequence. The light blue box highlights the mI exon, while the surrounding sequences are intronic. The amino acid sequence (corresponding to the Drosophila nucleotide sequences) is shown above the alignment. For positions with observed substitutions, it is noted below the alignment whether these are synonymous (s) or nonsynonymous (n). **(B)** To explore the roles of microexon mI ultraconservation in *Ubx* splicing control we engineered a series of *Ubx* minigene constructs so that they included wild type (wt) or mutated versions of microexon mI (mutA) in which the protein coding potential of the gene was maintained whilst the ultraconserved nucleotide sequence of mI was disrupted by means of synonymous mutations (red). Approximate positions of splicing primers *Ubx*_E1F (forward) and *Ubx*_3’U (reverse) and expression primers expF/R (forward/reverse) are indicated. **(C)** Experiments in Drosophila Schneider 2 (S2) cells. Semi-quantitative RT-PCR analysis of wild type and mutA *Ubx* minigenes expressed in S2 cells reveals distinct patterns of *Ubx* mRNA splicing where the mutA minigene construct shows a marked reduction of *Ubx* Ia isoform production. Ubx.AS refers to the signal detected with primers Ubx_E1F and Ubx_3’R (see panel B) which detects all alternative splicing variants of the gene; Ubx.exp denotes signal amplified with primers expF/R (see panel B) which are positioned in the 3’ exon, a constitutive segment of Ubx mRNAs. **(D)** Expression of *Ubx* wild type and *Ubx* mutA minigenes in the Drosophila embryo. We produced HA-tagged UAS versions of wt and mutA *Ubx* minigenes (see A) and generated independent transgenic UAS-lines with insertions in identical chromosomal loci by means of site-specific recombination. The resulting UAS-*Ubx* lines (wt and mutA) were crossed with the *elav-gal4* (*elav*) driver to express *Ubx* transgenes selectively within the developing embryonic nervous system. Note that expression patterns obtained with anti-HA antibodies in the embryonic central nervous system (CNS) and peripheral nervous system (PNS) were identical across genotypes confirming comparable gene expression conditions. **(E)** Semi-quantitative RT-PCR analysis of wt and mutA *Ubx* minigene expression in the embryonic Drosophila nervous system reveals effects of mI on *Ubx* splicing control. In line with the results obtained in S2 cells (see C) we observed that the mutA minigene produced a reduced amount of *Ubx* Ia isoform when compared to its wild type counterpart. (See C for definition of labels Ubx_AS and Ubx.exp and text for further details). [Statistical analyses: **P<0.01 and *P<0.05 obtained in one-tailed T-test (p-value S2 cells= 0.0035 (**); p-value embryos= 0.0318 (*). Error bars indicate standard error of the mean]. [HA: haemagglutinin tag; B, *Ubx* B-element; mI, microexon I; mII, microexon II].

**REFERENCES**

Adams MD, Celniker SE, Holt RA et al. 2000. The genome sequence of Drosophila melanogaster. Science. 287**:**2185-2195

Ahituv N, Zhu Y, Visel A, Holt A, Afzal V, Pennacchio LA, Rubin EM. 2007. Deletion of ultraconserved elements yields viable mice. PLoS Biol. 5**:**e234

Altschul SF, Gish W, Miller W, Myers EW, Lipman DJ. 1990. Basic local alignment search tool. J Mol Biol. 215**:**403-410

Artero RD, Akam M, Perez-Alonso M. 1992. Oligonucleotide probes detect splicing variants in situ in Drosophila embryos. Nucleic Acids Res. 20**:**5687-5690

Ashburner M, Ball CA, Blake JA et al. 2000. Gene ontology: tool for the unification of biology. The Gene Ontology Consortium. Nat Genet. 25**:**25-29

Bailey TL. 2011. DREME: motif discovery in transcription factor ChIP-seq data. Bioinformatics. 27**:**1653-1659

Bejerano G, Pheasant M, Makunin I, Stephen S, Kent WJ, Mattick JS, Haussler D. 2004. Ultraconserved elements in the human genome. Science. 304**:**1321-1325

Berghoff EG, Clark MF, Chen S, Cajigas I, Leib DE, Kohtz JD. 2013. Evf2 (Dlx6as) lncRNA regulates ultraconserved enhancer methylation and the differential transcriptional control of adjacent genes. Development. 140**:**4407-4416

Birnbaum RY, Patwardhan RP, Kim MJ et al. 2014. Systematic dissection of coding exons at single nucleotide resolution supports an additional role in cell-specific transcriptional regulation. PLoS Genet. 10**:**e1004592

Bischof J, Maeda RK, Hediger M, Karch F, Basler K. 2007. An optimized transgenesis system for Drosophila using germ-line-specific phiC31 integrases. Proc Natl Acad Sci U S A. 104**:**3312-3317

Boffelli D, Nobrega MA, Rubin EM. 2004. Comparative genomics at the vertebrate extremes. Nat Rev Genet. 5**:**456-465

Bomze HM, Lopez AJ. 1994. Evolutionary conservation of the structure and expression of alternatively spliced Ultrabithorax isoforms from Drosophila. Genetics. 136**:**965-977

Brand AH, Perrimon N. 1993. Targeted gene expression as a means of altering cell fates and generating dominant phenotypes. Development. 118**:**401-415

Brooks AN, Aspden JL, Podgornaia AI, Rio DC, Brenner SE. 2011. Identification and experimental validation of splicing regulatory elements in Drosophila melanogaster reveals functionally conserved splicing enhancers in metazoans. Rna. 17**:**1884-1894

Calin GA, Liu CG, Ferracin M et al. 2007. Ultraconserved regions encoding ncRNAs are altered in human leukemias and carcinomas. Cancer Cell. 12**:**215-229

Catucci I, Verderio P, Pizzamiglio S et al. 2009. SNPs in ultraconserved elements and familial breast cancer risk. Carcinogenesis. 30**:**544-545; author reply 546

Celniker SE, Dillon LA, Gerstein MB et al. 2009. Unlocking the secrets of the genome. Nature. 459**:**927-930

Chen CT, Wang JC, Cohen BA. 2007. The strength of selection on ultraconserved elements in the human genome. Am J Hum Genet. 80**:**692-704

Chiang CW, Liu CT, Lettre G et al. 2012. Ultraconserved elements in the human genome: association and transmission analyses of highly constrained single-nucleotide polymorphisms. Genetics. 192**:**253-266

Cunningham F, Amode MR, Barrell D et al. 2015. Ensembl 2015. Nucleic Acids Res. 43**:**D662-669

de la Calle-Mustienes E, Feijoo CG, Manzanares M, Tena JJ, Rodriguez-Seguel E, Letizia A, Allende ML, Gomez-Skarmeta JL. 2005. A functional survey of the enhancer activity of conserved non-coding sequences from vertebrate Iroquois cluster gene deserts. Genome Res. 15**:**1061-1072

de Navas LF, Reed H, Akam M, Barrio R, Alonso CR, Sanchez-Herrero E. 2011. Integration of RNA processing and expression level control modulates the function of the Drosophila Hox gene Ultrabithorax during adult development. Development. 138**:**107-116

Dellino GI, Tatout C, Pirrotta V. 2002. Extensive conservation of sequences and chromatin structures in the bxd polycomb response element among Drosophilid species. Int J Dev Biol. 46**:**133-141

Drake JA, Bird C, Nemesh J et al. 2006. Conserved noncoding sequences are selectively constrained and not mutation cold spots. Nat Genet. 38**:**223-227

Drosophila 12 Genomes Consortium. 2007. Evolution of genes and genomes on the Drosophila phylogeny. Nature. 450**:**203-218

Eden E, Navon R, Steinfeld I, Lipson D, Yakhini Z. 2009. GOrilla: a tool for discovery and visualization of enriched GO terms in ranked gene lists. BMC Bioinformatics. 10**:**48

Edgar RC. 2004. MUSCLE: multiple sequence alignment with high accuracy and high throughput. Nucleic Acids Res. 32**:**1792-1797

Feng J, Bi C, Clark BS, Mady R, Shah P, Kohtz JD. 2006. The Evf-2 noncoding RNA is transcribed from the Dlx-5/6 ultraconserved region and functions as a Dlx-2 transcriptional coactivator. Genes Dev. 20**:**1470-1484

Glazov EA, Pheasant M, McGraw EA, Bejerano G, Mattick JS. 2005. Ultraconserved elements in insect genomes: a highly conserved intronic sequence implicated in the control of homothorax mRNA splicing. Genome Res. 15**:**800-808

Glazov EA, Pheasant M, Nahkuri S, Mattick JS. 2006. Evidence for control of splicing by alternative RNA secondary structures in Dipteran homothorax pre-mRNA. RNA Biol. 3**:**36-39

Griffith OL, Montgomery SB, Bernier B et al. 2008. ORegAnno: an open-access community-driven resource for regulatory annotation. Nucleic Acids Res. 36**:**D107-113

Harmston N, Baresic A, Lenhard B. 2013. The mystery of extreme non-coding conservation. Philos Trans R Soc Lond B Biol Sci. 368**:**20130021

Hatton AR, Subramaniam V, Lopez AJ. 1998. Generation of alternative Ultrabithorax isoforms and stepwise removal of a large intron by resplicing at exon-exon junctions. Mol Cell. 2**:**787-796

Heckman KL, Pease LR. 2007. Gene splicing and mutagenesis by PCR-driven overlap extension. Nat Protoc. 2**:**924-932

International Glossina Genome Initiative. 2014. Genome sequence of the tsetse fly (Glossina morsitans): vector of African trypanosomiasis. Science. 344**:**380-386

Kellis M, Patterson N, Endrizzi M, Birren B, Lander ES. 2003. Sequencing and comparison of yeast species to identify genes and regulatory elements. Nature. 423**:**241-254

Kent WJ, Sugnet CW, Furey TS, Roskin KM, Pringle TH, Zahler AM, Haussler D. 2002. The human genome browser at UCSC. Genome Res. 12**:**996-1006

Kern AD, Barbash DA, Mell JC, Hupalo D, Jensen A. 2015. Highly constrained intergenic Drosophila ultraconserved elements are candidate ncRNAs. Genome biology and evolution.

Koch R, Ledermann R, Urwyler O, Heller M, Suter B. 2009. Systematic functional analysis of Bicaudal-D serine phosphorylation and intragenic suppression of a female sterile allele of BicD. PLoS One. 4**:**e4552

Kritsas K, Wuest SE, Hupalo D, Kern AD, Wicker T, Grossniklaus U. 2012. Computational analysis and characterization of UCE-like elements (ULEs) in plant genomes. Genome Res. 22**:**2455-2466

Lampe X, Samad OA, Guiguen A, Matis C, Remacle S, Picard JJ, Rijli FM, Rezsohazy R. 2008. An ultraconserved Hox-Pbx responsive element resides in the coding sequence of Hoxa2 and is active in rhombomere 4. Nucleic Acids Res. 36**:**3214-3225

Langmead B, Trapnell C, Pop M, Salzberg SL. 2009. Ultrafast and memory-efficient alignment of short DNA sequences to the human genome. Genome Biol. 10**:**R25

Lareau LF, Inada M, Green RE, Wengrod JC, Brenner SE. 2007. Unproductive splicing of SR genes associated with highly conserved and ultraconserved DNA elements. Nature. 446**:**926-929

Lee TI, Jenner RG, Boyer LA et al. 2006. Control of developmental regulators by Polycomb in human embryonic stem cells. Cell. 125**:**301-313

Lin MF, Kheradpour P, Washietl S, Parker BJ, Pedersen JS, Kellis M. 2011. Locating protein-coding sequences under selection for additional, overlapping functions in 29 mammalian genomes. Genome Res. 21**:**1916-1928

Lin Z, Ma H, Nei M. 2008. Ultraconserved coding regions outside the homeobox of mammalian Hox genes. BMC Evol Biol. 8**:**260

Liz J, Portela A, Soler M, Gomez A, Ling H, Michlewski G, Calin GA, Guil S, Esteller M. 2014. Regulation of pri-miRNA processing by a long noncoding RNA transcribed from an ultraconserved region. Mol Cell. 55**:**138-147

Lopez AJ, Hogness DS. 1991. Immunochemical dissection of the Ultrabithorax homeoprotein family in Drosophila melanogaster. Proc Natl Acad Sci U S A. 88**:**9924-9928

Makunin IV, Kolesnikova TD, Andreyenkova NG. 2014. Underreplicated regions in Drosophila melanogaster are enriched with fast-evolving genes and highly conserved noncoding sequences. Genome biology and evolution. 6**:**2050-2060

Makunin IV, Shloma VV, Stephen SJ, Pheasant M, Belyakin SN. 2013. Comparison of ultra-conserved elements in drosophilids and vertebrates. PLoS One. 8**:**e82362

Manoli DS, Foss M, Villella A, Taylor BJ, Hall JC, Baker BS. 2005. Male-specific fruitless specifies the neural substrates of Drosophila courtship behaviour. Nature. 436**:**395-400

Megy K, Emrich SJ, Lawson D et al. 2012. VectorBase: improvements to a bioinformatics resource for invertebrate vector genomics. Nucleic Acids Res. 40**:**D729-734

Mestdagh P, Fredlund E, Pattyn F et al. 2010. An integrative genomics screen uncovers ncRNA T-UCR functions in neuroblastoma tumours. Oncogene. 29**:**3583-3592

Mlodzik M, Fjose A, Gehring WJ. 1985. Isolation of caudal, a Drosophila homeo box-containing gene with maternal expression, whose transcripts form a concentration gradient at the pre-blastoderm stage. The EMBO journal. 4**:**2961-2969

Ni JZ, Grate L, Donohue JP, Preston C, Nobida N, O'Brien G, Shiue L, Clark TA, Blume JE, Ares M, Jr. 2007. Ultraconserved elements are associated with homeostatic control of splicing regulators by alternative splicing and nonsense-mediated decay. Genes Dev. 21**:**708-718

Nielsen MM, Tehler D, Vang S, Sudzina F, Hedegaard J, Nordentoft I, Orntoft TF, Lund AH, Pedersen JS. 2014. Identification of expressed and conserved human noncoding RNAs. Rna. 20**:**236-251

Nusslein-Volhard C, Wieschaus E. 1980. Mutations affecting segment number and polarity in Drosophila. Nature. 287**:**795-801

Papp B, Muller J. 2006. Histone trimethylation and the maintenance of transcriptional ON and OFF states by trxG and PcG proteins. Genes Dev. 20**:**2041-2054

Pennacchio LA, Ahituv N, Moses AM et al. 2006. In vivo enhancer analysis of human conserved non-coding sequences. Nature. 444**:**499-502

Poitras L, Yu M, Lesage-Pelletier C et al. 2010. An SNP in an ultraconserved regulatory element affects Dlx5/Dlx6 regulation in the forebrain. Development. 137**:**3089-3097

Quinlan AR, Hall IM. 2010. BEDTools: a flexible suite of utilities for comparing genomic features. Bioinformatics. 26**:**841-842

R Development Core Team. (2011) R: A Language and Environment for Statistical Computing. <http://www.R-project.org/>.

Ramaswami G, Li JB. 2014. RADAR: a rigorously annotated database of A-to-I RNA editing. Nucleic Acids Res. 42**:**D109-113

Reed HC, Hoare T, Thomsen S, Weaver TA, White RA, Akam M, Alonso CR. 2010. Alternative splicing modulates Ubx protein function in Drosophila melanogaster. Genetics. 184**:**745-758

Reneker J, Lyons E, Conant GC, Pires JC, Freeling M, Shyu CR, Korkin D. 2012. Long identical multispecies elements in plant and animal genomes. Proc Natl Acad Sci U S A. 109**:**E1183-1191

Richards S, Liu Y, Bettencourt BR et al. 2005. Comparative genome sequencing of Drosophila pseudoobscura: chromosomal, gene, and cis-element evolution. Genome Res. 15**:**1-18

Rogulja-Ortmann A, Picao-Osorio J, Villava C, Patraquim P, Lafuente E, Aspden J, Thomsen S, Technau GM, Alonso CR. 2014. The RNA-binding protein ELAV regulates Hox RNA processing, expression and function within the Drosophila nervous system. Development. 141**:**2046-2056

Ryoo HD, Marty T, Casares F, Affolter M, Mann RS. 1999. Regulation of Hox target genes by a DNA bound Homothorax/Hox/Extradenticle complex. Development. 126**:**5137-5148

Ryu T, Seridi L, Ravasi T. 2012. The evolution of ultraconserved elements with different phylogenetic origins. BMC Evol Biol. 12**:**236

Sandelin A, Bailey P, Bruce S, Engstrom PG, Klos JM, Wasserman WW, Ericson J, Lenhard B. 2004. Arrays of ultraconserved non-coding regions span the loci of key developmental genes in vertebrate genomes. BMC Genomics. 5**:**99

Scott JG, Warren WC, Beukeboom LW et al. 2014. Genome of the house fly, Musca domestica L., a global vector of diseases with adaptations to a septic environment. Genome Biol. 15**:**466

Siepel A, Bejerano G, Pedersen JS et al. 2005. Evolutionarily conserved elements in vertebrate, insect, worm, and yeast genomes. Genome Res. 15**:**1034-1050

Stark A, Lin MF, Kheradpour P et al. 2007. Discovery of functional elements in 12 Drosophila genomes using evolutionary signatures. Nature. 450**:**219-232

Steffen PA, Ringrose L. 2014. What are memories made of? How Polycomb and Trithorax proteins mediate epigenetic memory. Nat Rev Mol Cell Biol. 15**:**340-356

Stergachis AB, Haugen E, Shafer A et al. 2013. Exonic transcription factor binding directs codon choice and affects protein evolution. Science. 342**:**1367-1372

Teytelman L, Thurtle DM, Rine J, van Oudenaarden A. 2013. Highly expressed loci are vulnerable to misleading ChIP localization of multiple unrelated proteins. Proc Natl Acad Sci U S A. 110**:**18602-18607

Thomsen S, Azzam G, Kaschula R, Williams LS, Alonso CR. 2010. Developmental RNA processing of 3'UTRs in Hox mRNAs as a context-dependent mechanism modulating visibility to microRNAs. Development. 137**:**2951-2960

Visel A, Prabhakar S, Akiyama JA, Shoukry M, Lewis KD, Holt A, Plajzer-Frick I, Afzal V, Rubin EM, Pennacchio LA. 2008. Ultraconservation identifies a small subset of extremely constrained developmental enhancers. Nat Genet. 40**:**158-160

Viturawong T, Meissner F, Butter F, Mann M. 2013. A DNA-centric protein interaction map of ultraconserved elements reveals contribution of transcription factor binding hubs to conservation. Cell Rep. 5**:**531-545

Woolfe A, Goodson M, Goode DK et al. 2005. Highly conserved non-coding sequences are associated with vertebrate development. PLoS Biol. 3**:**e7

Yang R, Frank B, Hemminki K et al. 2008. SNPs in ultraconserved elements and familial breast cancer risk. Carcinogenesis. 29**:**351-355

Yang Z. 1997. PAML: a program package for phylogenetic analysis by maximum likelihood. Comput Appl Biosci. 13**:**555-556
